# Insights for Oxidative Stress and mTOR Signaling in Myocardial Ischemia/Reperfusion Injury under Diabetes

**DOI:** 10.1155/2017/6437467

**Published:** 2017-02-19

**Authors:** Dajun Zhao, Jian Yang, Lifang Yang

**Affiliations:** ^1^Department of Cardiac Surgery, Xijing Hospital, The Fourth Military Medical University, Xi'an 710032, China; ^2^Department of Anesthesiology, Xi'an Children's Hospital, Xi'an 710003, China

## Abstract

Diabetes mellitus (DM) displays a high morbidity. The diabetic heart is susceptible to myocardial ischemia/reperfusion (MI/R) injury. Impaired activation of prosurvival pathways, endoplasmic reticulum (ER) stress, increased basal oxidative state, and decreased antioxidant defense and autophagy may render diabetic hearts more vulnerable to MI/R injury. Oxidative stress and mTOR signaling crucially regulate cardiometabolism, affecting MI/R injury under diabetes. Producing reactive oxygen species (ROS) and reactive nitrogen species (RNS), uncoupling nitric oxide synthase (NOS), and disturbing the mitochondrial quality control may be three major mechanisms of oxidative stress. mTOR signaling presents both cardioprotective and cardiotoxic effects on the diabetic heart, which interplays with oxidative stress directly or indirectly. Antihyperglycemic agent metformin and newly found free radicals scavengers, Sirt1 and CTRP9, may serve as promising pharmacological therapeutic targets. In this review, we will focus on the role of oxidative stress and mTOR signaling in the pathophysiology of MI/R injury in diabetes and discuss potential mechanisms and their interactions in an effort to provide some evidence for cardiometabolic targeted therapies for ischemic heart disease (IHD).

## 1. Introduction

Diabetes mellitus (DM) is a major risk factor for ischemic heart disease (IHD) [1]. The alteration of glucose metabolism leads to cardiac structural and functional perturbations, including left ventricular (LV) dysfunction, cardiac hypertrophy, and myocardial interstitial fibrosis. A number of diabetic subjects suffer from the impairments of diastolic dysfunction in an early stage without overt cardiovascular symptoms [2–4]. Cardiac hypertrophy is originally a compensatory response to pathological overload stress. However, the persistent DM-induced hypertrophy ultimately becomes maladaptive since it evolves into cardiac dysfunction, and finally develops into heart failure [5–7]. Hyperglycemia directly increases cardiac fibroblast and vascular smooth muscle cell proliferation and is associated with endothelial dysfunction, resulting in microvascular injury and hemodynamic alteration, which contribute to the vulnerability of tissue ischemia injury [8, 9]. Myocardial salvage after reperfusion may be limited by deleterious changes in the microcirculation of ischemic tissue [10]. All these pathophysiologic changes in the diabetic heart lead to a susceptibility to ischemia/reperfusion (I/R) injury [9, 11]. Consequences of increased cellular apoptosis and inflammation are present in the diabetic heart subjected to I/R injury [12–14]. It is truly different from the normotensive mechanisms since metabolic abnormalities and alteration of oxidative stress and autophagy. Among all these factors, oxidative stress and the mammalian target of rapamycin (mTOR) signaling are two critical ones [15, 16].

Oxidative stress is defined as an imbalance between free radicals production and destruction, which leads to multiple negative effects on cellular metabolism. mTOR kinase is also necessary for normal regulation of cardiac structure and cardiometabolic homeostasis. It promotes mitochondrial function in response to insulin resistance and affects cardiac energy deprivation and ischemia [17, 18]. Both of them participate in the pathogenesis and progression of myocardial ischemia/reperfusion (MI/R) injury under diabetes, acting as key regulators of cardiometabolism and cardiac function. However, the relationship between oxidative stress and mTOR signaling is complicated, since mTOR not only modulates oxidative stress but is also affected by reactive oxygen species (ROS) activation. In this review, we will focus on the role of oxidative stress and mTOR signaling in the pathophysiology of I/R injury in the diabetic heart and highlight their current interactions in an effort to provide some evidence for the potential cardiometabolic targeted therapies for IHD.

## 2. The Vulnerability of Diabetic Heart Subjected to MI/R Injury

DM severely damages cardiac energy homeostasis, leading to the cardiac dysfunction. It is well recognized that populations associated with DM were more likely to develop IHD and their long-term outcome is worsened [19]. Importantly, physical or pharmacologic ischemic preconditioning (IPC) and ischemic postconditioning (I-post) actions are ineffective under diabetic conditions [20–22], suggesting that the diabetic heart may be resistant to common cardioprotections.

### 2.1. Impaired Activation of Prosurvival Pathways

In the diabetic heart, the alteration of reperfusion injury salvage kinase (RISK) signaling significantly suppressed the cardioprotective effects of IPC [23, 24]. Studies demonstrated that glycogen synthase kinase-3*β* (GSK-3*β*) was activated by insulin resistance, thus inhibiting the prosurvival pathway of the phosphoinositide-3 kinase- (PI3k-) Akt signaling and the Janus-activated kinase- (JAK-) transcription 3 (STAT3) signaling, finally blunting the cardioprotective effects of I-post [25, 26]. Moreover, our previous study proved that adiponectin (APN) resistance existed in the diabetic cardiomyocytes and impaired APN's cardioprotection against MI/R injury [27]. APN resistance led to the dysfunctional APN-AMP-activated protein kinase (AMPK) axis and blocked the AMPK-independent antiperoxide/antinitration pathway, increasing the vulnerability of diabetic cardiomyocytes to I/R injury [27, 28].

### 2.2. Endoplasmic Reticulum (ER) Stress

Disturbed cardiometabolic homeostasis facilitates ER stress. The unfolded protein response (UPR) was proved to be involved in the pathogenesis of DM [29, 30]. Miki et al. demonstrated that DM-induced ER stress augmentation enhanced the mitochondrial permeability transition pore (mPTP) opening and increased mitochondrial calcium overload via inhibition of extracellular regulated MAP kinase (ERK) 1/2- GSK-3*β* pathway [31]. In contrast, suppression of ER stress could reduce myocardial infarction (MI) size in high fat diet- (HFD-) induced type 2 diabetes mellitus (T2DM) [32]. Our recent study found that preconditioning of C1q/TNF-related protein (CTRP) 9, a newly identified homologous of APN, protected the diabetic heart against I/R injury by reducing ER stress and inflammatory response [33].

### 2.3. Increased Basal Oxidative State and Impaired Antioxidant Signaling

Hyperglycemia enhances oxidative stress, promotes profibrogenic genes expression, and aggravates MI/R injury [34, 35]. ROS accumulation not only results from overproduction of free radicals, but also may be a consequence of decreased free radicals scavenger systems, including superoxide dismutase (Cu/Zn-SOD and Mn-SOD), catalase (CAT), and glutathione peroxidase (GPx) [36]. Cardiac expression of GPx levels is reduced in the diabetic apolipoprotein E-deficient mice [37]. Meanwhile, attempts to attenuate I/R injury using enzymatic and nonenzymatic antioxidants have not been universally successful in DM [38, 39].

### 2.4. Autophagy and mTOR Signaling

Autophagy is a cellular degradation pathway that crucially mediates cardiometabolism. It has been demonstrated that autophagy was required for IPC via mTOR signaling and Parkin-dependent pathway [40, 41]. However, mitochondrial biogenesis is impaired in the diabetic heart, following the alteration of autophagic activity. Hyperglycemia largely inhibited cardiac autophagosome and autolysosome formation by modulating mTOR-ULK1 signaling [42]. It deteriorated the cardioprotection of remote IPC (rIPC) because of the increase in nitrative stress and inhibition of autophagy via activation of mTOR signaling [43, 44].

## 3. The Role of Oxidative Stress in MI/R Injury under Diabetes

**Figure 1:**
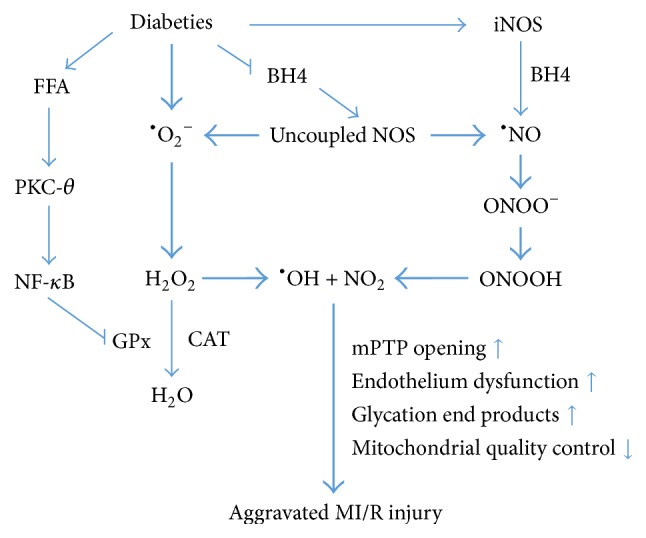
DM-induced higher basal oxidative state plays a master role in the progression of cardiometabolic disorders and negatively affects the MI/R injury. In this state, ROS and RNS accumulate dramatically. They initiate the reaction of ^*∙*^OH in parallel with the ONOO^−^/ONOOH generation, which becomes strong cytotoxic oxidant and causes oxidative damage and nitration. These then lead to endothelium dysfunction, formation of advanced glycation end products, and alteration of the mitochondrial quality control, all contributing to the deleterious MI/R injury in diabetic hearts. Free fatty acid (FFA); protein kinase C-*θ* (PKC-*θ*); nuclear factor of kappa light polypeptide gene (NF-*κ*B); superoxide (^*∙*^O_2_^−^); hydrogen peroxide (H_2_O_2_); glutathione peroxidase (GPx); catalase (CAT); hydroxyl (^*∙*^OH); tetrahydrobiopterin (BH4); nitric oxide synthase (NOS); inducible NOS (iNOS); nitric oxide (^*∙*^NO); peroxynitrite (ONOO^−^); peroxynitrous acid (ONOOH); nitrogen dioxide (NO_2_); mitochondrial permeability transition pore (mPTP).

Oxidative stress is regarded as an imbalance between the generation and elimination of free radicals due to increased ROS and/or inadequate antioxidant defenses [45]. It develops directly or indirectly from hyperglycemia, hyperlipidemia, and insulin resistance under DM [15, 46] and in turn, disturbs metabolic hemostasis and impairs cardiac function. When available in appropriate amounts, free radicals act as signal transduction molecules while in large excess, they lead to DNA degeneration, lipid oxidation and membrane protein degeneration. However, in the diabetic heart, insulin resistance increases cardiomyocytes fatty acid oxidation together with a reduction of prostacyclin synthesis and endothelial nitric oxide (eNOS) synthase activity [47]. These changes lead to generation of ROS and reactive nitrogen species (RNS), endothelium dysfunction, formation of advanced glaciation end products, and alteration of the mitochondrial quality control, all of which contribute to the deleterious MI/R injury under diabetes [48]. Thus, DM-induced oxidative stress can be a primary component that initiates the onset and progression of cardiac dysfunction in MI/R injury (Figure 1).

### 3.1. ROS and RNS Production

ROS are a group of short-lived, low-molecular-weight compounds derived from variety of reactions oxygen undergoes, including superoxide (^*∙*^O_2_^−^), hydroxyl (^*∙*^OH), hydrogen peroxide (H_2_O_2_), and hypochlorous acid (HOCl). The generation of ROS in the heart is few under physiologic conditions. ^*∙*^O_2_^−^ leakages from mitochondrial electron transport chains (ETC) and soon be catalyzed into less cytotoxic H_2_O_2_ by SOD catalyzes, then finally be converted into water and molecular oxygen by either CAT or GPx system [45]. However, the homeostasis of cardiac oxidative state would be broken under DM since the generation of ^*∙*^O_2_^−^ increased markedly. The accumulated ^*∙*^O_2_^−^ is highly diffusible and damages cardiomyocytes [49]. H_2_O_2_ is more likely converted to ^*∙*^OH other than scavenged by CAT or GPx [50]. Moreover, hyperglycemia increases cardiac free fatty acid (FFA) levels, which extensively leads to a great rise of ROS formation and a reduction of GPx by activating nuclear factor of kappa light polypeptide gene (NF-*κ*B) [51, 52] and its upstream mediator protein kinase C-*θ* (PKC-*θ*) [53]. ROS enhances mPTP opening, contributing to myocardial contractile dysfunction and tissue damage in ischemia-reperfused rat hearts [54].

Diabetic myocardial RNS production is also greatly increased, including radicals nitric oxide (^*∙*^NO) and nitrogen dioxide (^*∙*^NO_2_^−^). The rapid reaction of superoxide with nitric oxide (NO) forms a highly reactive intermediate, peroxynitrite (ONOO^−^), under MI/R injury. With increased intracellular acidification, ONOO^−^ becomes more protonated to form peroxynitrous acid (ONOOH), which then rapidly turns into nitrogen dioxide (NO_2_) and ^*∙*^OH. The ONOO^−^/ONOOH becomes strong cytotoxic oxidant and causes oxidative damage and nitration, which contributes in parallel with the reaction of ^*∙*^OH generation during MI/R [45].

### 3.2. Uncoupled NOS

Diabetic mice exhibited increased risk of aggravated MI/R injury primarily because of impaired NO bioavailability. ONOO^−^ may uncouple eNOS via oxidation of tetrahydrobiopterin (BH4), thus leads to further superoxide generation and an enhanced NO depletion [55]. However, reduced availability of BH4 was identified in diabetic rat vessels and endothelial cells. DM-induce NADPH increase further predisposes the heart to NOS uncoupling and ONOO^−^ generation [56]. Maalouf et al. demonstrated that* S*-glutathionylation uncoupled eNOS and subsequently impaired endothelium-dependent vasodilation under oxidative stress [57]. Moreover, inducible NOS (iNOS) is activated in DM by inflammatory mediators, which makes iNOS uncoupling a predominant contributor for oxidative/nitrosative stress in diabetic myocardium [58].

### 3.3. Disturbing the Mitochondrial Quality Control

Mitochondria are the major sites of ROS production (0.2% to 2% of total oxygen taken by cells). These ROS can be scavenged by mitochondrial quality control to keep the mitochondria functional [59]. However, in the diabetic heart, mitochondrial quality control is damaged together with impaired mitochondrial respiratory capacity, leading to a dramatic accumulation of ROS [15]. Importantly, increased mitochondrial H_2_O_2_ emission then damages DNA, proteins, and lipid in membrane components and finally results in mitochondrial dysfunction [60]. The myocardium of db/db mice exhibited increased mitochondrial H_2_O_2_ generation, and overproduction of mitochondrial ROS occurring in conjunction with augmented electron delivery from increased fatty acid oxidation [51]. Taken together, these studies suggest that mitochondrial quality control regulates cellular oxidative stress, while, if damaged, oxidative stress in turn might affect mitochondrial dysfunction under DM.

## 4. The Dual Role of mTOR Signaling in MI/R Injury under Diabetes

mTOR is a 289 kDa serine/threonine kinase that crucially mediates energy metabolism [61]. It has two distinct multiprotein complexes, mTOR complex 1 (mTORC1) and mTOR complex 2 (mTORC2) [62–64]. mTORC1 regulates cellular homeostasis, stress responses, energy metabolism and autophagy by relying on the regulatory associated protein of mTOR (Raptor). In contrast, mTORC2 treats rapamycin-insensitive companion of mTOR (Rictor) as the component rather than Raptor and controls cell growth, survival, migration, and cell cycle progression [65]. mTOR kinase is necessary for normal regulation of cardiac structure and cardiometabolism. It also takes part in the maintenance of normal microvascular barrier function and endothelial permeability. However, the role of mTOR signaling in MI/R injury is still controversial. Researchers have found both cardioprotective and cardiotoxic effects of mTOR signaling when using its inhibitor-rapamycin or transgenic animals [66]. Besides, there is a complicated interplay between mTOR signaling and oxidative stress (Table 1).

### 4.1. Cardiotoxic Effects of mTOR Signaling

Chronic increase of mTORC1 activity in T2DM causes insulin resistance, which contributes to hyperinsulinemia and hyperglycemia [79–81]. Evidences showed that mTORC1 was activated in the hearts of obese and diabetic animals during reperfusion, increasing the vulnerability of MI/R injury. In HFD-induced obesity mice, cardiac autophagosome formation was decreased, accompanied by cardiac dysfunction, which could be reversed by rapamycin (2 mg/kg, intraperitoneal injection, i.p.) and worsened by genetic APN disruption [82]. mTOR phosphorylates the mammalian homologue of autophagy related gene 13 (Atg13) and the mammalian Atg1 homologues UNC-51-like kinase 1 (ULK1) and ULK2 to prevent the progression of autophagy. Sciarretta et al. confirmed that rapamycin administration (1 mg/kg, i.p.) or partial mTOR deletion significantly reduced infarct size after ischemia through the restoration of autophagy [83]. Our previous studies also found that hypertension-induced mTOR activation altered cardiac morphology, function, and autophagy, which could be rescued by cardiac-specific overexpression of metallothionein [84, 85].

Importantly, activation of mTORC1 other than mTOR2 signaling affects cardiac metabolism and the susceptibility to ischemia injury [67]. A patient-level meta-analysis of randomized trials showed that selective activation of mTORC2 with concurrent inhibition of mTORC1 decreased cardiomyocytes apoptosis and tissue damage after MI [86]. It seems that different complex of mTOR performs different cardiac functions. Another cardiotoxic mTOR effect is altering STAT3 signaling pathway in the diabetic heart. Das et al. found that inhibition of mTOR by rapamycin (0.25 mg/kg, i.p.) before ischemia reduced I/R-induced MI in CD-1 mice via activating the JAK2-STAT3 signaling [87]. This was further proved in cardiac-specific STAT3-deficient mice [88].

### 4.2. Cardioprotections of mTOR Signaling

There are four major mTOR-related cardioprotective pathways: (1) insulin-mediated PI3K/Akt/mTOR signaling pathway; (2) GSK-3*β* inhibition signaling pathway; (3) mTOR-dependent angiogenesis signaling pathway; (4) mTORC2 activation signaling pathway. Cardiac PI3K/Akt causes insulin-stimulated glucose uptake and induces acute mTOR activation, thus improving cardiomyocytes survival and function [73]. Studies found that the PI3K/Akt/mTOR signaling pathway provided efficient cardioprotection against I/R injury induced by insulin [74]. Aoyagi et al. further observed that cardiac-specific transgenic mice overexpressing mTOR suppressed I/R-induced inflammation and necrosis, inhibited cardiac fibrosis in adverse LV remodeling in diet-induced obesity mice [89]. They demonstrated that Akt phosphorylation was higher in mTOR overexpressed mice than WT mice under HFD conditions and it was unlikely that mTOR's cardioprotective effects were mediated through autophagic activity. Zhang et al. also demonstrated that Lin28a overexpression protected against MI/R injury in diabetic mice through the insulin-PI3K-mTOR pathway [90].

mTOR's cardioprotection required the inhibition of GSK-3*β* to reduce the reperfusion injury through mTORC1 hyperactivation [85]. During periods of I-post, mTOR prevents cardiomyocytes apoptosis via mTOR-dependent GSK-3*β* inhibition mechanisms. mTORC1 regulates mPTP opening and promotes mitochondrial biogenesis, which may favor cardiac recovery after MI/R and promote the upregulation of antioxidant genes via the activation of proliferator-activated receptor *γ* coactivator-1*α* (PGC-1*α*) [85, 91, 92].

The altered lipid metabolism induced by insulin resistance results in a propensity for microvascular barrier dysfunction, accelerated atherosclerosis, increased vessel wall reactivity, and plaque complications. Angiogenesis is an important component of cardioprotection against I/R injury, which has been proved to be mechanically via mTOR-dependent pathway. Inhibition of mTOR signaling by rapamycin (2 *μ*M) for 1 h leads to subsequent impaired angiogenesis in aortic endothelial cells [75]. Loss of mTOR activity by rapamycin (5–10 ng/mL) also blocks endothelial proliferation and angiogenesis [93] as well as the proliferation of endothelial progenitor cells ex vivo [94]. Hypoxia activates the mTOR pathway to promote angiogenesis and cell proliferation [93, 95]. mTOR activation enhances the activity of HIF1*α* by inhibiting proteolytic degradation, resulting in elevated VEGF expression. This effect is reversible by rapamycin (25 nM for human umbilical vein endothelial cells and 50 nM for HEK293 cells) [68, 69].

Study found that the cardioprotective effects mediated by mTOR overexpression were partly dependent on mTORC2 activation, which was beneficial to cardiomyocytes survival against I/R injury as well as chronic ischemic remodeling [96]. Considering that mTORC2 is rapamycin-insensitive, it is reasonable that using rapamycin to inhibit mTORC1 activity also presents cardioprotective actions against MI/R injury [97]. However, there was still little understanding of the complexity of mTORC2's regulation and its roles in cardiac functions.

### 4.3. Interactions between Oxidative Stress and mTOR Signaling

Cardiac mTOR is considered as an important regulator of oxidative stress by promoting mitochondrial biogenesis and oxidative metabolism through Ying-Yang 1- (YY1-) PGC-1*α* pathway [92]. Meanwhile, mTOR modulates autophagy, increases mitochondrial clearance and protects cardiomyocytes from oxidative stress-induced toxicity [72, 98] through the activation of protein kinase B (PKB) [99]. In contrast, other studies found that in cardiac mTOR disrupted mice, fatty acid oxidation is significantly decreased, whereas glucose oxidation is increased [100]. mTOR regulates oxidative stress-induced endothelium dysfunction. Inhibition of mTORC1 either with rapamycin or by S6K1 silencing recouples eNOS function, improves NO production, and inhibits O_2_^−^ generation in the rat aortas [71]. mTOR also modulates cardiac fibrosis in the models of post-MI remodeling and cardiac hypertrophy [70, 101, 102] while treatment with rapamycin reduced ROS production in the myofibroblasts.

On the other hand, oxidative stress regulates mTORC1 ordinarily. ROS production contributes to the inhibition of GSK-3*β* and mTOR signaling [85]. Alternative origins of ROS, such as NADPH oxidase, may as well provoke mTOR activation and subsequent impair autophagy [76]. An intriguing link between peroxisomes, oxidative stress and autophagy has been recently described. Peroxisomal ROS has been shown to suppress mTORC1 activity, in models of the tuberous sclerosis complex signaling node TSC1 and TSC2 proteins [77, 103]. In contrast, Vigneron et al. found that, in the isolated-perfused mouse heart, IPC protected against I/R injury via inhibition of GSK-3*β* and a constant opening of mitoKATP with ROS generation to activate the mTOR pathway and induce cardioprotection [104].

## 5. Potential Cardiometabolic Target against Diabetic MI/R Injury

Although animal studies have found potential regulator aiming at oxidative stress or mTOR signaling under experimental diabetic conditions, clinical studies are still disappointing. Thus, new therapeutic targets as well as efficient cardioprotections against DM-induced MI/R injury are urgently needed.

### 5.1. Metformin

It is well recognized that metformin could reduce cardiovascular end points of T2DM independently from its glucose-lowering effects. Administration of metformin significantly attenuates I/R injury via relieving ER stress [105] and activating of AMPK-eNOS prosurvival pathway in both nondiabetic and diabetic mice [105, 106]. However, further research demonstrated that metformin effectively attenuated LV hypertrophy and dysfunction by activating mTOR, p70S6K (Thr389), and S6 phosphorylation in both wild-type and AMPK*α*2 KO mice, suggesting that metformin attenuated myocardial mTOR signaling independently of AMPK*α*2 activation [107]. Metformin reduces ROS generation and ameliorates oxidative stress-induced apoptosis and inflammation in cardiomyocytes [108] and endothelial cells [109]. It also protects against I/R-induced myocardial fibrosis by inhibiting fibrotic factors, including TGF-*β*1, TNF-*α* and basic fibroblast growth factor (bFGF) in the circulation and the myocardium [110, 111]. As a routine oral agent for T2DM, metformin might be a potential pharmacological therapeutic target to protect against MI/R injury under diabetes on the regulation of cardiac oxidative stress and mTOR signaling.

### 5.2. Sirtuin 1 (Sirt1)

Sirt1 is a member of Sirtuins family [77]. It controls cellular processes and maintains metabolic homeostasis by reducing apoptosis, attenuating inflammation, and modulating oxidative stress [112, 113]. It is a critical regulator in DM-induced MI/R injury. Sirt1-mediated PGC-1*α* activation could directly respond to H_2_O_2_-induced oxidative stress on the regulation of glutathione GPx1, CAT, and Mn-SOD [114]. Overexpression of Sirt1 inhibited oxidative stress and reduced MI/R injury via modulating eNOS activity under diabetic condition [113]. There is a crosstalk between AMPK, Sirt1 and mTOR signaling in the regulation of oxidative stress and cardiomyocytes autophagy [115–117]. Sirt1 deacetylates FoxO3a while mTORC1 can inhibit FoxO-mediated transcription of antioxidant gene targets, including the antioxidants Mn-SOD and catalase [116]. Meanwhile, Sirt1 positively regulates transcription of Rictor, activating the mTORC2 signaling by triggering a cascade of Akt and FoxO phosphorylation. Sirt1 deficiency mice performed increased ROS production and impaired mTORC2 signaling, leading to insulin resistance that could be largely reversed with antioxidant treatment [78]. Considering its specific functions in modulating oxidative stress, mTOR signaling, and mitochondrial dysfunction perturbed in the diabetic heart, Sirt1 may be a promising novel therapeutic target for MI/R injury under DM.

### 5.3. CTRP9

CTRP9 is a newly found APN paralog. It protects against obesity and T2DM through anti-inflammation and antiapoptotic actions. Increasing the circulating CTRP9 level is a beneficial action against HFD-induced obesity and glucose intolerance [118], whereas CTRP9-deficiency mice performed exacerbated insulin resistance [119]. Importantly, CTRP9 performs cardioprotective effects via inhibition of oxidative stress. Kambara et al. demonstrated that administration of exogenous CTRP9 inhibited oxidative stress, attenuated cardiomyocytes apoptosis, and suppressed inflammatory reactions in the ischemic heart [120, 121]. Su et al. observed the same results in the HFD-induced T2DM mice, implicating that differing from APN, there is no CTRP9 resistance in DM [122]. Our recent finding proved that CTRP9 protected the diabetic heart against I/R injury by reducing ER stress and inflammatory response [33]. Interestingly, compared to general pharmacologic antioxidants, the amount of cardiac endogenous CTRP9 is abundant, much higher than its expression in adipocytes and circulation, suggesting that CTRP9 may be a novel cardiokine. These findings indicate that CTRP9 may also be a potential therapeutic target for diabetic cardiac complications.

## 6. Conclusions

It is well established that DM aggravates MI/R injury and diabetic IHD patients experience worse clinical outcomes. Oxidative stress and mTOR signaling are master mediators of cardiometabolism and MI/R injury. ROS and RNS accumulation induces cardiomyocytes damage by direct oxidation of proteins, reactive lipid peroxidation products, and interaction with DNA. Uncoupling NOS tigers oxidation/nitration reaction and disturbing the mitochondrial quality control causes mitochondrial dysfunction. These may be mechanisms of oxidative stress impairing the diabetic heart.

When turning to mTOR signaling, it is still controversial to clearly understand the role of mTOR signaling in MI/R injury under DM since both cardioprotective and cardiotoxic effects were observed in vivo and in vitro. The conflicted outcomes could be explained by the following. (1) There is different duration of rapamycin treatment [123]. Study demonstrated that inhibition of mTORC1 before ischemia reduced the size of MI while rapamycin was not cardioprotective if administered before the reperfusion phase [87]. Moreover, different duration of rapamycin treatment contributes to the alteration of metabolic homeostasis. Houde et al. found that administration of rapamycin for two weeks could enhance the insulin level, leading to a glucose intolerance and insulin resistance in mice. However, more than six weeks treatment could improve insulin sensitivity [124]. (2) There is different phosphorylation site of mTORC1. mTORC1 predominately phosphorylated the specific site encompassing 4E-BP1 (T37) and (T46) that are rapamycin resistant. However, mTORC1 could phosphorylated S6K1 (T389), which is rapamycin sensitive under conditions. (3) There are degrees of mTOR activation in the regulation of autophagy. Yu et al. demonstrated that mTOR signaling was inhibited during autophagy initially, but reactivated with prolonged autophagy. The progress was autophagy-dependent and required the degradation of autolysosomal products. The enhanced mTOR activity in verse attenuated autophagy [125]. (4) There are different cardiac functions of mTORC1 and mTORC2. mTORC1 presents both beneficial and detrimental effects on MI/R injury while mTORC2 show mostly cardioprotective actions as its cellular survival functions [96, 97]. (5) There are inescapable defects of loss-of-function animal models. Conventional ablation of mTOR in mice results in embryonic death [126–128] while cardiac-specific mTOR knockout mouse also shows fatal, dilated cardiomyopathy [64]. Other deletions of mTOR downstream molecules including Raptor and S6K1 may partially inhibit mTOR signaling and also be detrimental since not only the maladaptive but also the physiological functions of the kinase are ablated.

**Figure 2:**
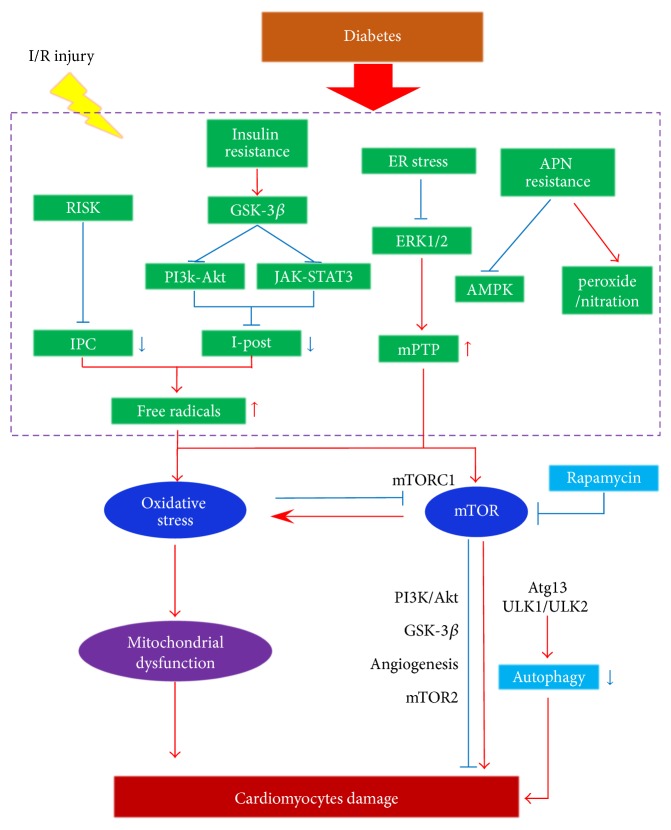
The diabetic heart is susceptible to MI/R injury. Impaired activation of prosurvival pathways, endoplasmic reticulum (ER) stress, increased basal oxidative state, and decreased antioxidant defense and autophagy may render diabetic hearts to be more vulnerable to MI/R injury and be resistant to ischemic preconditioning (IPC) or ischemic postconditioning (I-post). Oxidative stress and mTOR signaling crucially regulate cardiometabolism, affecting MI/R injury under diabetes. Reperfusion injury salvage kinase (RISK); phosphoinositide-3 kinase (PI3k); glycogen synthase kinase-3*β* (GSK-3*β*); signal transducer and activator of transcription (STAT); autophagy related gene 13 (Atg13); mammalian Atg1 homologues UNC-51-like kinase (ULK); Janus kinase 2 (JAK2); extracellular regulated MAP kinase (ERK).

The interplay between oxidative stress and mTOR signaling is complicated, since mTOR not only modulates oxidative stress but also is affected by oxidative stress activation [129]. However, it is unlikely that these fully explain what occurs in the diabetic heart, considering its complicated pathophysiological conditions. Further studies using appropriate in vivo models of DM are needed (Figure 2).

No therapeutic strategy has yet been demonstrated clinically effective against cardiac injury in diabetic population. Antihyperglycemic agent metformin and newly found free radicals scavengers, Sirt1 and CTRP9, may serve as promising pharmacological cardiometabolic targeted therapeutic genes.

## Figures and Tables

**Table 1:** Cardioprotective and cardiotoxic effects of mTOR signaling in MI/R injury under diabetes.

Effect of mTOR	Study	Animal model	Interventions	Outcomes
Cardioprotective	Glazer et al. [67]	Transgenic mice	Overexpression of cardiac mTOR	Overexpression of cardiac mTOR reduced mortality in the acute phase and preserved cardiac function in the chronic phase after transient ischemia in vivo

Cardioprotective	Land and Tee [68]	Transgenic mice	Overexpression of cardiac mTOR	mTOR-Tg mice performed better cardiac function recovery and had less of the necrotic markers CK and LDH subjected to I/R injury in high fat diet-induced obesity

Cardioprotective	Park et al. [69]	Diabetic mice induced by STZ	Rapamycin (5 mg/kg i.v.) 10 min before I/R	Lin28a overexpression increased p-mTOR and p-p70s6k expression in myocardium exposed to I/R injury in diabetic mice while inhibition of mTOR reduced Lin28a's cardioprotective effects

Cardioprotective	Schenkel et al. [70]	C57BL/6 mice	Torin1 (i.p.) immediately after MI with a short 2-day follow-up treatment to inhibit both mTORC1 and mTORC2 Rictor and Raptor siRNA (i.v.) to selectively inhibit mTORC1 or mTORC2 PRAS40 siRNA (i.v.) to inhibit mTOR1	Inhibition of both mTORC1 and mTORC2 with Torin1 led to increased cardiomyocyte apoptosis and tissue damage after MI. Predominant mTORC1 signaling by suppression of mTORC2 similarly increased cardiomyocyte apoptosis and tissue damage after myocardial infarction. In comparison, preferentially shifting toward mTORC2 signaling by inhibition of mTORC1 with PRAS40 led to decreased cardiomyocyte damage after MI

Cardioprotective	Tanguy et al. [50]	Neonatal rat ventricular cardiomyocytes	Rapamycin: 50 nM Adenovirus overexpressing mTOR	Inhibition of mTOR by rapamycin antagonized high glucose-induced inhibition of autophagy and enhanced cardiomyocyte death, while adenovirus-mediated overexpression of mTOR was sufficient to block autophagic flux regardless of glucose concentrations

Cardioprotective	Rajapakse et al. [71]	Human umbilical vein endothelial cells (HUVECs)	Rapamycin: 25 nM	mTOR activation enhances the activity of HIF1*α* by inhibiting proteolytic degradation, resulting in elevated VEGF expression

Cardioprotective	Chong et al. [72]	Human endothelial cells	Rapamycin: 5–10 ng/mL	Loss of mTOR blocks endothelial proliferation and angiogenesis as well as the proliferation of endothelial progenitor cells ex vivo

Cardiotoxic	Yao et al. [73]	APN knockout mice	Rapamycin (2 mg/kg, i.p.)	Rapamycin reversed APN deficiency-induced drop of fat oxidation in high fat diet feeding

Cardiotoxic	Si et al. [74]	Transgenic mice	Conditional mTOR knockout mice	Inhibition of mTORC1 reduced endoplasmic reticulum stress, thereby reducing cardiomyocytes death

Cardiotoxic	Lemaître et al. [75]	CD-1 mice	Rapamycin (0.25 mg/kg, i.p.)	Inhibition of mTOR by rapamycin before ischemia reduced I/R-induced myocardial infarction via activating the JAK2 signal transducer and activator of transcription 3 (STAT3) signaling pathway

Cardiotoxic	Fourcade et al. [76]	Transgenic mice	Cardiac-specific knockout Raptor to inhibit mTORC1 in vivo	In cardiac mTORC1 disrupted mice, fatty acid oxidation is significantly decreased, whereas glucose oxidation is increased subjected to transverse aortic constriction (TAC)

Cardiotoxic	Maiese et al. [77]	Male WKY rats and HUVECs	Recombinant adenoviral (rAd) expressing short hairpin RNA (shRNA), S6K1 to inhibit mTORC1	Inhibition of mTORC1/S6K1 signaling protected endothelial dysfunction related to eNOS uncoupling in vivo and in vitro

Cardiotoxic	Wang et al. [78]	AMPK*α*2 knockout mice	Metformin (100 mg/kg/day, gavage) for 3 weeks	Administration of metformin was effective in attenuating TAC-induced LV remodeling in both wild-type and AMPK*α*2 knockout mice and reduced p-mTOR at Ser2448 and its downstream target p-p70S6K at Thr389
